# Secure Telemedicine System Design for COVID-19 Patients Treatment Using Service Oriented Architecture

**DOI:** 10.3390/s22030952

**Published:** 2022-01-26

**Authors:** Asadullah Shaikh, Mana Saleh Al Reshan, Adel Sulaiman, Hani Alshahrani, Yousef Asiri

**Affiliations:** College of Computer Science and Information Systems, Najran University, Najran 61441, Saudi Arabia; asshaikh@nu.edu.sa (A.S.); msalreshan@nu.edu.sa (M.S.A.R.); aaalsulaiman@nu.edu.sa (A.S.); yasiri@nu.edu.sa (Y.A.)

**Keywords:** SOA security architecture, Tele-COVID SOA security, telemedicine security, COVID-19 patients treatment

## Abstract

The coronavirus pandemic, also known as the COVID-19 pandemic, is an ongoing virus. It was first identified on December 2019 in Wuhan, China, and later spread to 192 countries. As of now, 251,266,207 people have been affected, and 5,070,244 deaths are reported. Due to the growing number of COVID-19 patients, the demand for COVID wards is increasing. Telemedicine applications are increasing drastically because of convenient treatment options. The healthcare sector is rapidly adopting telemedicine applications for the treatment of COVID-19 patients. Most telemedicine applications are developed for heterogeneous environments and due to their diverse nature, data transmission between similar and dissimilar telemedicine applications is a difficult task. In this paper, we propose a Tele-COVID system architecture design along with its security aspects to provide the treatment for COVID-19 patients from distance. Tele-COVID secure system architecture is designed to resolve the problem of data interchange between two different telemedicine applications, interoperability, and vendor lock-in. Tele-COVID is a web-based and Android telemedicine application that provides suitable treatment to COVID-19 patients. With the help of Tele-COVID, the treatment of patients at a distance is possible without the need for them to visit hospitals; in case of emergency, necessary services can also be provided. The application is tested on COVID-19 patients in the county hospital and shows the initial results.

## 1. Introduction

The COVID-19 pandemic was discovered in Wuhan, Hubei China, in December 2019, which has later spread to several nations. As of 12 November 2021; almost 252,097,350 (COVID-19 Dashboard https://www.arcgis.com/apps/dashboards/85320e2ea5424dfaaa75ae62e5c06e61 (accessed on 30 September 2021)) humans have been infected with COVID-19 and the death percentage is approximately 2.1%. The COVID-19 is declared a worldwide pandemic where the human death ratio has increased. With an increasing number of COVID-19 patients, hospitals were out of space, and even in a few countries were not accepting new patients due to the lack of wards and space available. It has been also discovered that COVID-19 is not harmful until or unless it is spread to the lungs [1]. Therefore, the chances are lower for asymptomatic cases to transmit COVID-19 from one human to another [2].

The COVID-19 first case was detected in late December 2019 [3] and slowly and gradually, the number of infections increased up to July 2020. A sudden hike was seen in cases until mid-September 2020 the scientists called this the first wave of COVID-19 [4]. Consequently, a slight decline in COVID-19 cases was noticed during mid-September 2020 but in early October 2020, a sudden increase diverted the attention of the whole world and this could indicate the start of a ‘Second wave’ [5] that can be considerably more dangerous than the first wave [6].

It has been also discovered that the physiological effects of COVID-19 are more dangerous than having the disease itself [7]. Due to the physiological effect, many COVID-19 patients rush to hospitals out of fear. Nevertheless, it is not mandatory to hospitalize COVID-19 patients if the disease is not severe.

The COVID-19 is still a mystery, and no one knows about its ending time. Therefore, to overcome the different waves of COVID-19 pandemic, telemedicine systems deliver effective and prominent solutions to stop its spread. Telemedicine applications provide an efficient platform to establish secure communication between health professionals and patients. To overcome the crisis and public health emergencies, several telemedicine solutions are proposed [8]. One of the popular methods that have been strictly followed to stop the spread of COVID-19 infection is ‘social distancing’ which means to decrease human-to-human contact. Considering the concept of ‘social distancing’, the telemedicine application provides the platform that allows physicians to examine patients remotely. Telemedicine frameworks have been created and used to establish communication between clinical experts and patients utilizing the online platform, i.e., web-based and mobile applications [9]. The demand for telemedicine web-based and mobile applications is increasing rapidly due to the COVID-19 pandemic. Telemedicine applications are extremely helpful to provide an online communication platform reducing physical attendance for unnecessary appointments to receive medical support [10].

Service Oriented Architecture (SOA) is a method for software design that makes software components reusable by utilizing service interfaces used as a common communication language on a network. A service is an independent unit of software components, or a set of functionalities, intended to do a particular job, for example, recovering determined data or executing an activity. It contains the code and information integrations necessary to do a complete job. Subsequently, SOA integrates software parts that have been independently deployed to communicate and form software applications across various frameworks. Before SOA came into utilization in the last part of the 1990s, interfacing an application to services housed in another framework was a difficult process including deep point-to-point integration—connectivity, routing, interpretation of information models, and so forth which then, at that point must be reproduced by software developers with each new task. In a service-oriented design style, services are utilizing a system of “free coupling”. This is a method of interconnecting parts (additionally called “components”) in a framework or network with the goal that they can pass data or facilitate a business cycle while decreasing the conditions between them. This, creates a new application [10,11]. We studied and analyzed almost 50 articles on SOA in telemedicine and concluded that SOA and its security provides unique designs that can be used in telemedicine applications for COVID-19 patients since security is the core component of any telemedicine system. The system was designed in order to provide a service that could be reused, with improved efficiency in making new applications. Moreover, the author of [12] proposed a new application called Expert Medical Information System (EMIS). The proposed application can be used to diagnose different diseases using expert systems. SOA was applied by researchers in [8] by proposing a new application called tele-wound. The proposed application was aimed at utilizing experts’ input without depending on the patient’s location. Nevertheless, SOAMOH is another application proposed in [13]. The proposed system leveraging an SOA-based platform that they refer to as a service-oriented architecture for m-healthcare. SOAMOH was envisioned to facilitate the providence of healthcare services to people anytime and anywhere using their mobile devices connected via wireless networking technologies. However, the proposed approach faced confidentiality issues in some regions because health records are regarded as confidential. Further, the mobile system’s network is very volatile and has a lot of incompatibilities that could present challenges to the SOAMOH platform’s full utility in telemedicine.

**Figure 1:**
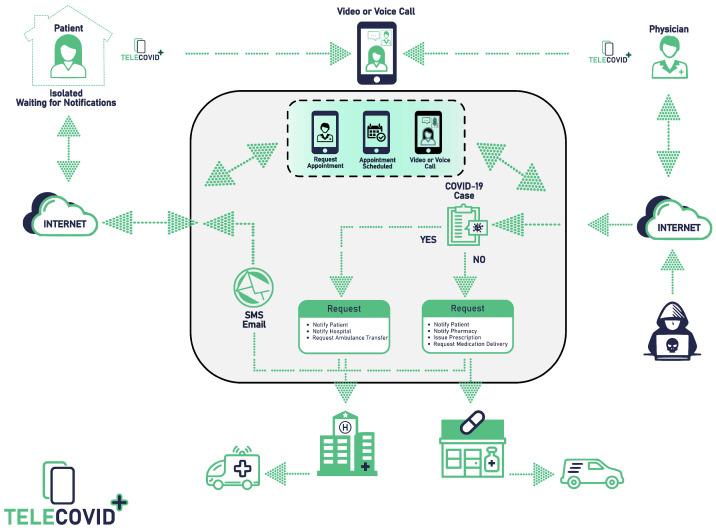
Basic structure of Tele-COVID using SOA [14].

We have proposed Tele-COVID which is a telemedicine application to examine and provide suitable treatment to COVID-19 patients from a distance [14]. The implementation of Tele-COVID is uniquely designed in SOA that helps to abstain from the issues related to vendor lock-in, interoperability, and data interchange. Tele-COVID facilitates COVID patients to seek advice from expert physicians without visiting the hospital. In case of emergency, Tele-COVID can provide necessary services like ambulance service to transport the patient from home to hospital and physicians can prescribe the medication for the infected patients. This SOA-based telemedicine application presents a unique architecture which consists of four Sections (1) Patient (2) Physician/Doctor (3) Pharmacy, and (4) Hospital. The first section represents the patient’s access to the physician where suspected COVID patient can request an appointment with the physician through Tele-COVID. The second section is based on different doctors who are accessible online through the Tele-COVID application to take a clinical history from the patient and choose the seriousness of the case. The third is about pharmacy, where the physician can notify the pharmacy to deliver the required medication to the patient’s house without visiting the hospital. The final section of the Tele-COVID is deployed in the hospital with the emergency clinic to organize the pickup for the patients in case hospitalization is required. Figure 1 shows the basic structure of Tele-COVID using SOA.

Several works are published and proposed to describe the necessity of telemedicine applications and their ability in emergencies. However, in the COVID-19 crisis, the important element is to stop the transmission of the virus, and this can be achieved by ‘social distancing’. Telemedicine applications are developed and deployed to overcome the complexities of communication between patients and medical staff using online and mobile services [9]. Currently, telemedicine applications have become essential during the COVID-19 pandemic. These applications are the only platform that will create safe interaction between patients and hospitals with the help of telemedical services. This will reduce physical attendance and redundant appointments to receive medical treatment [10]. The proposed secure architecture is different from present architectures as it emerges with SOA for every single component. Consequently, the integrated functions of Tele-COVID are extendable to any current telemedicine application without any additional implementation strategies. This ensures that there will be no gap in data exchange from one telemedicine application to another.

The implementation of SOA security in telemedicine is hardly found in the literature. Therefore, in this paper, we propose a security architecture for Tele-COVID using the secure techniques of SOA. The proposed solution is based on security aspects for all four sections, i.e., (1) Patient (2) Physician/Doctor (3) Pharmacy, and (4) Hospital. To arrange the security aspects for all sections, our proposed secure engineering configuration depends on Shared Secret Key (SSK) security for validation and approval. When each end/section establishes the communication with each other, a message will be encrypted through the digester and Message Authentication Code (MAC). Once the message is received to another side, it can be decrypted using digester and Message Integrity Authentication (MIA).

The rest of the paper is structured as follows. Section 2 presents the related work. Section 3 focuses on the security requirements for the Tele-COVID application while Section 4 explores the research method used in the study. Section 5 presents the security architecture of Tele-COVID with four main parts, i.e., the implementation of SOA in Tele-COVID and the role of SOA in Tele-COVID. Section 6 discusses the security implementation in Tele-COVID. Discussions and evaluation of Tele-COVID SOA security architecture is elaborated on in Section 7. Finally, Section 8 offers conclusions and future directions.

## 2. Related Work

### 2.1. SOA in Telemedicine

The research [15] was one of the first articles that were concerned with the use of SOA to resolve the issue of interoperability in telemedicine systems. Therefore, the authors focused on using telemedicine to provide medical solutions to patients at their place. With the use of SOA, the designed architecture provided a service that could be reused, improved efficiency in making new applications, and the developed application could model services separately without considering their implementation environment that made dissemination through any other service easy. Therefore, SOA supported the development of applications that had physical and data-type interoperability. The authors of [12] focused on EMIS. The expert system that is proposed in this research can support disease diagnostic decisions, and in this case, the researchers chose to use hemorrhagic dengue. Therefore, they managed to simulate the human expert, and through mobile computing, the healthcare professionals could have mobility out of the designated medical lefts. The authors successfully showed that EMIS’s architecture could provide services that put into account the electronic health records and expert systems. The service consumers under EMIS were healthcare practitioners. The service provider is composed of a Universal Description, Discovery, and Integration (UDDI) server, web services, medical process engine, configuration manager, configuration interface, expert system handler, expert system, knowledge base, and resource manager, resource, medical information manager, medical information, and security service.

Another example is in [8] where the researchers focus on SOA to avail the ideal architecture and propose a next-generation tele-wound application. The tele-wound is based on multimedia communication, and plain texts and pictures are proposed in this case. The architecture proposed aims at availing expert input irrespective of the patient’s location. The technological aspects of concern entail fast network, and therefore, the researchers suggested teleconferencing. Patients require having a mobile phone or a computer that has access to the tele-wound application. The doctor’s end also should have a device to monitor the patient’s condition. The researchers try to provide a solution to the highly complex nature of telemedicine and the use of SOA seems very promising as software continues to evolve to support more sophisticated applications.

The paper [13] looked at mobile healthcare systems as a solution to skyrocketing costs in healthcare providence. By integrating data from disparate sources, clinical decision-making processes would be improved. Issues such as time delays in emergency rooms would be solved. The authors suggest leveraging an SOA-based platform that they refer to as service-oriented architecture for m-healthcare (SOAMOH). SOAMOH was envisioned to facilitate the providence of healthcare services to people anytime and anywhere using their mobile devices connected via wireless networking technologies. The authors proposed an architecture that contained components such as body sensor networks that is wearable, an application-specific communication infrastructure, its service management, and a caregiver/practitioner and external service consumers. It is noted that SOAMOH had an advantage over other built applications in that it was designed and developed for service orientation. Therefore, SOAMOH was scalable and made it possible for use of new services and applications. However, SOAMOH utilization was faced with confidentiality issues in some regions because health records are regarded as confidential. Further, the mobile system’s network is volatile and has a lot of incompatibilities that could present challenges to the SOAMOH platform’s full utility in telemedicine.

The authors in [16] believe that by solving the interoperability issues, the management of information systems may eliminate technological, informational, and organizational barriers that affect service delivery. To resolve the issue of heterogeneity and complexity that results from the use of telemedicine and the need to work collaboratively, the authors suggested a Model-Driven Architecture (MDA) and SOA as an area of research that can be leveraged to provide information system solutions. Notably, telemedicine entails providing a solution to complex medical issues, and using both MDA and SOA would ensure sufficient exchange of information. These researchers show that MDA has been used successfully to track patient information in health clinic systems. However, they note that it has been a challenge meeting the needs of modularity, interoperability, and extensibility of the target health services. Therefore, the authors focused on availing several functionalities of telemedicine in form of web services. The researchers successfully showed that a joint approach can be attained that will entail the use of both SOA and MDA when developing telemedicine applications in developing countries. The approach can be utilized to cover many countries as interoperability of different applications is facilitated. Therefore, basing the functionality of key applications in telemedicine on the MDA concept, interoperability as the services are provided can be attained.

### 2.2. SOA Security

SOA security issues were noted in [17] about the existing configuration and that there was a deficiency in the methodology for SOA applications. Issues on how to configure security in the application development process were poorly defined, information for configuring security was unavailable, and it was difficult to understand the type of security that was required by only viewing the business requirements. Therefore, the researchers suggested an end-to-end security configuration using supporting services to offer the architects and assemblers the required help to generate complicated configuration files. The supporting technologies suggested were the model-driven security and pattern-based policy configuration. This was not to be a challenge because the SOA application consisted of assemblies of service components, and the inclusion of other service components was not affecting efficiency. Therefore, under the proposed methodology for configuring security based on “model-driven development”, a single developer is entrusted with the completion of the configuration. Configurations are written using certain tools that these developers (such as assemblers) do not fully understand. Since the tools are available and reducing the information about writing configurations, security assurances could be improved under SOA.

The authors in [18] noted that the challenges of managing the overarching problem of managing the non-functional requirements of SOA relate to the many ways a consumer can access the services offered. One of the primary requirements of the SOA is the Quality of Security Service (QoSS) because it has to include aspects of privacy. The authors managed to come up with the structure for the QoSS metadata and since this needed to be placed within an SOA component or application, it was referred to as service QoSS (SQoSS). The SOA security framework and the SQoSS were implemented with the use of .NET technologies. The researchers in [19] presented the Platform Independent Model for Service-Oriented Architectures (PIM4SOA). In the PIM4SOA model, there was an elaborate explanation of service, process, information, and quality of service. The authors illustrated the model that they had presented by giving a sample scenario in which it was applied in an online bookshop known as Ama-Zen. In this case, there was the use of CreditCard Web Service, and integrity and confidentiality aspects were highly prioritized. Through the case, the authors illustrated the Web Services Description Language (WSDL) definition (CreditCard) that had an embedded policy and how it was applied to operation messages. PIM4SOA was implemented under the Eclipse platform and facilities therefore this was an extendable and modular approach. Prospects into how this metamodel could be extended were provided.

Thirteen security snares that firms had to consider regarding the implementation of SOA as mentioned in [20]. First, firms had to address assumptions of the software vendor taking care of the security. Another issue related to not asking about security and due to limited knowledge, people in charge of security in a firm could ask the wrong questions. SOA could also come with discomforts that had to be ignored to prioritize software security. Other organizations had to assume that the chances of loopholes in SOA being utilized by malicious people were minimal. Other issues highlighted included believing that there is security for no apparent reason, misapplying vulnerability metrics, excessive trust in the vendors, ignoring immediate security when active, trusting someone else to handle security, giving up hope, and overemphasizing security. This article shoes doubts and speculations that surrounded SOA implementation.

It is suggested in [21] that business process modeling should be used as the layer in which security requirements are described and risks evaluated. Therefore, in this research, the researchers utilized the MDA as the approach for integrating their security goals and used the Business Process Model and Notation (BPMN) as their modeling language in the business process model. In their case scenario, they demonstrated their work using the “Online Student Information System” to show how necessary permissions can be assigned to particular learners to perform their different tasks. The authors conclude by noting that during the early stages of developing SOA applications, security is not incorporated due to issues with current general-purpose modeling languages and the lack of a clear definition of SOA’s security objectives.

In resource-sharing settings such as cloud computing and the internet of things (IoT), more applications based on service-oriented architecture target multiple users. Consequently, data privacy and data privacy protection issues always emerge. The authors [22] focused on a security model of “Public-Key Encryption (PKE) in the multi-user setting, selective opening security for receiver corruptions” and this model integrates aspects of security for PKE schemes in cases where there are compromised users. PKE constructions in SOA security are based on the fact that ciphertexts of some users can be opened. However, for those whose ciphertexts have not been opened, their messages should remain confidential. In a case where there is a receiver corruption, the hacker or the adversary can access even the secret keys that can be utilized in decrypting the opened ciphertexts. In this research, the authors focused on PKE constructions that achieve security and simulation-based SOA (SIM-SOA) security (for receiver corruptions) that operate under chosen-ciphertext attacks.

The researchers in [23] focused on data provenance as it relates to data quality, processing, and routing into the SOA system. Data provenance is important because it helps the decision-makers decide on the quality of the data. They came up with SOA’s new framework for data provenance. This framework had features such as agile data classification and dynamic analyses. The framework also allowed various data collection strategies such as actor-based and message-based. However, some issues related to multilevel data provenance security, and one approach these authors recommend for implementing multilevel security is to ensure that a security level is assigned before the service becomes active. Also, a duplicate copy of workflow or service to each level can be assigned. The SOA data provenance framework that was proposed in this study had been used for a food company in enterprise Service-Oriented Production Planning Systems. In their proposed framework, the authors focused on the reliability, integrity, and security of data during its routing in an SOA system.

The major challenge in the fight against SARS-CoV-2 is the poor diagnostic tools and infrastructure for early detection of positive COVID-19 cases, majorly in impoverished and rural areas of developing countries. Therefore, a fast, cheaper, and efficient machine learning diagnostic-based system is warranted. A factorial experiment was designed to assess the Machine Learning (ML) functionality with an X-ray of chest images for pneumonia, healthy, and COVID-19-infected patients. The evaluation of the ML technique was based on the categorization of metrics such as the area under the receiver operating features curve (AUROC), accuracy, sensitivity, F1-score, and specificity. The experimental design generated the mean and confidence interval for the different predictive capabilities of the diverse ML techniques used. Among the algorithms for learning, the vector machines support and the random forest had the best performance. The investigation indicated that ML techniques had the capability to identify patients infected with COVID-19. The results showed improved values for specificity and sensitivity, reducing the false-negative and false-positive rates. Additionally, the low computation resources used in this study made it possible for these techniques to be used in rural and impoverished areas [24].

Researchers in [25] highlighted the important issues facing telemedicine privacy use, client data storage, limited internet accessibility, and poor infrastructure. The paper proposes for the Indian government to facilitate the adoption of essential telemedicine competencies in both undergraduate and postgraduate programs. The importance of drawing telemedicine programs into the current education system is to improve the training of professionals, which would ultimately improve the level of healthcare services rendered. Additionally, with numerous concerns over client data and storage privacy, the importance of proper security protection cannot be overstated. In a bid to ensure privacy, the authors note that India’s Parliament tabled a Bill in December 2019 which led to the formation of a Data Protection Authority. Thanks to the bill, it is now possible to impose huge penalties when the law concerning data storage is violated. Accessing patient data is now only possible if these patients consent to it. The authors conclude that positive external aid such as investment in telemedicine could significantly lead to more equitable and accessible healthcare in low and middle-income countries (LMIC).

The related work discussed above demonstrates SOA with several services. SOA security, in general, has been broadly addressed in research and development. However, secure SOA architecture for telemedicine systems is hardly found in the literature. Tele-COVID architecture is equipped with services that help interoperability, data interchange, and automatic transmission of data from similar or dissimilar telemedicine architecture to another. Moreover, the application can facilitate COVID-19 patients round the clock.

## 3. Security Requirements in Tele-COVID

In mobile and web applications, the SOA approach is widely used to develop several components for secure communication [26]. There are various messaging protocols to establish secure communication from one end to another. However, Web Service (WS) security provides Simple Object Access Protocol (SOAP) messaging to apply security measures especially when it comes to message confidentiality, integrity, and message authentication. Also, SOAP messages can be transmitted using encryption techniques. This sort of security gives an adaptable plan to security models like Secure Sockets Layer (SSL) and Kerberos. Nevertheless, it facilitates trust domains, security tokens, including signatures with encryption techniques. To exchange secure messages between one end to another, WS security tokens (digester) are used which will be shared from the requester and provider. WS security is flexible in use, however, its setup in real-world examples is tricky for users. To the best of our knowledge CRUD, (Create, Read, Update, and Delete) operations are not sufficiently applied along with SOA security. Therefore, Tele-COVID is equipped with CRUD operation along with SOA security tokens which we also called digesters. There are a total of four security requirements for Tele-COVID that will be discussed in the next subsection

- Transmission of secure messages and establishment of secure communication between Patient and Physician.
- Transmission of secure messages and establishment of secure communication between Physician and Pharmacy.
- Transmission of secure messages and establishment of secure communication between Physician and Hospital.
- Transmission of secure messages and establishment of secure communication between Pharmacy and Patient.

### Security Implementation at Physician and Patient End

**Figure 2:**
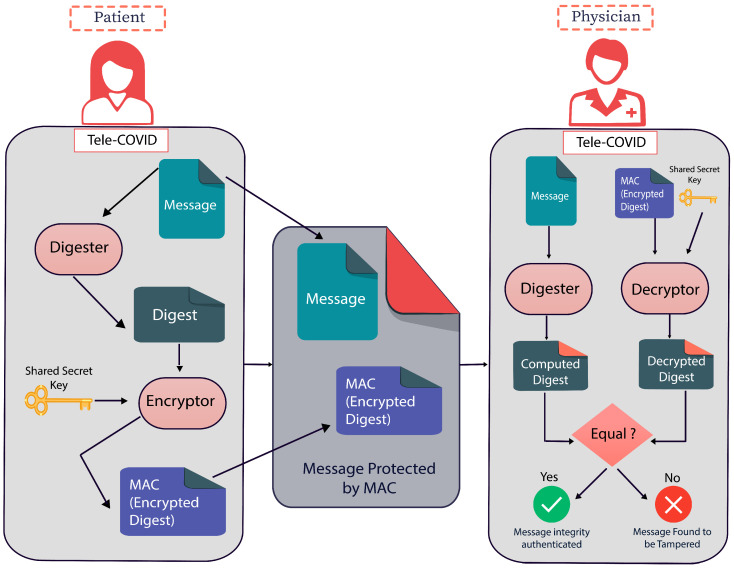
SOA secure message communication between patient and physician.

In this section, we describe the basic architecture of security implementation between physician and patient end. This security model will be applied to all four security requirements of Tele-COVID. First, the suspected COVID-19 patients will fill the questionnaires through a home test to set up the seriousness of the infection. Second, the patient may ask for an appointment. Third, the physician will affirm the appointment and the patient will be advised through the Tele-COVID application. Fourth, the patient will begin his conversation about the symptoms with the doctor through secure communication. Figure 2 shows the SOA secure message communication between patient and physician, where the message is sent through a digester and is encrypted using MAC.

Once the message is delivered which will be in encrypted form, the physician will decrypt it using SSK and if the message is not tempered it will be decrypted using MIA. Alternatively, a message will be discarded if found tempered. This process will be continued until the end of the conversation between patient and physician. Fifth, the conversation between two ends can be established using a video or voice meeting. Sixth, the doctor will choose if the patient can be treated at home. On the off chance that the patient does not have serious COVID-19 symptoms, then the doctor will encourage the patient to remain at home and prescribe him some essential medicine if required. Seventh, the clinical prescription will be delivered to the pharmacy automatically where Tele-COVID is already installed and the pharmacy will transport the medicine through a delivery service managed by them. The communication between pharmacy and physician will be undertaken the same way as between physician and patient.

Finally, the suspected COVID-19 patient could be in critical condition, therefore, the doctor will give a solicitation to the hospital to send a rescue vehicle to move the patient from home to the COVID-19 special ward for further treatment.

## 4. Research Method

An intensive literature review approach was integrated in parallel with preliminary bibliographical research. Qualitative and conceptual approaches were used at the later stage. A comparison between our proposed secure architecture and existing architecture was conducted based on data collected from a survey. As a result, a comparative analysis of various circumstances relevant to a wide variety of companies to address various issues such as data integration, interoperability, and vendor locking in the domain of telemedicine systems. For the quantitative approach, an effort was made to adhere to a methodology that involved improving the telemedicine architectural system to:

- Identifies the related security aspect of telemedicine systems.
- Examines specific security methods for Tele-COVID applications for all four ends.
- Proposes a security architecture for Tele-COVID that will address the issues of data integration, interoperability and vendor locking.
- Transmits secure encrypted messages from patient to physician and from physician to the hospital.

**Figure 3:**
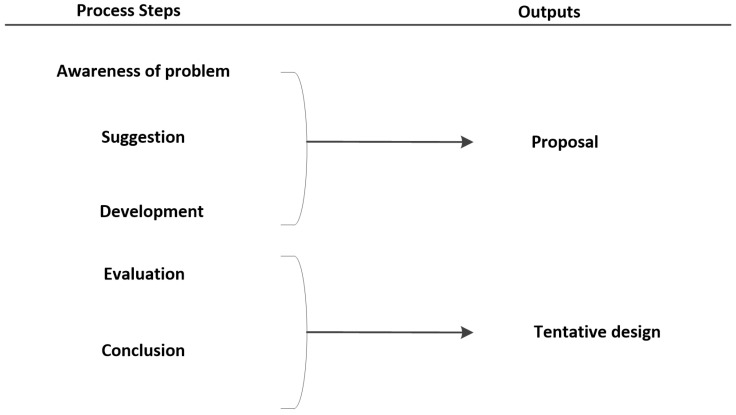
Design research methodology.

Additionally, the practice of design research was adopted; this is sometimes referred to as improvement research, as it is a widely used approach for problem-solving across different domains. It was necessary to design a telemedicine architecture solution that addresses the issues of data integration, interoperability, and vendor locking. We adopted the following procedures of design research, as shown in Figure 3.

**a**  Process Steps: Techniques that contribute to creating innovation through design research are:
  - Awareness of Problems: Security in web-based and mobile applications is currently discussed everywhere, and several methods are being applied for the security methods. However, SOA security is one of the major important elements is being implemented in several telemedicine applications. Thus, we need to make sure that our mobile application and web-based application meet the minimum-security requirements as the awareness step.
  - Suggestion: We provide the solution based on SOA security that is represented in Figure 2, where the patient will send the message that will be digested. After the digestion, the message will be encrypted, and through Tele-COVID, the message will be deployed to the physician end. Consequently, the physician can decrypt the message using the shared secret key.
  - Development: In this part of the process, we applied the SOA security in our web-based application, the Tele-COVID application which stores the encrypted messages into databases.
  - Evaluation: The secure Tele-COVID is currently functional in four ends that are hospital, patient, physician, and pharmacy.
  - Conclusion: To check the security whether it is implemented in all four ends.
**b**  Outputs: There will be a unique output in each of the steps. The awareness of the problem and suggestion belongs to the proposal. In case, a researcher finds any errors in the development stage, it can go back to the second step or the third step of the methodology, otherwise in this methodology, we do not have a return mechanism. The targeted output is obtaining a secure Tele-COVID application or system.

### Flow Chart of the Proposed Method

**Figure 4:**
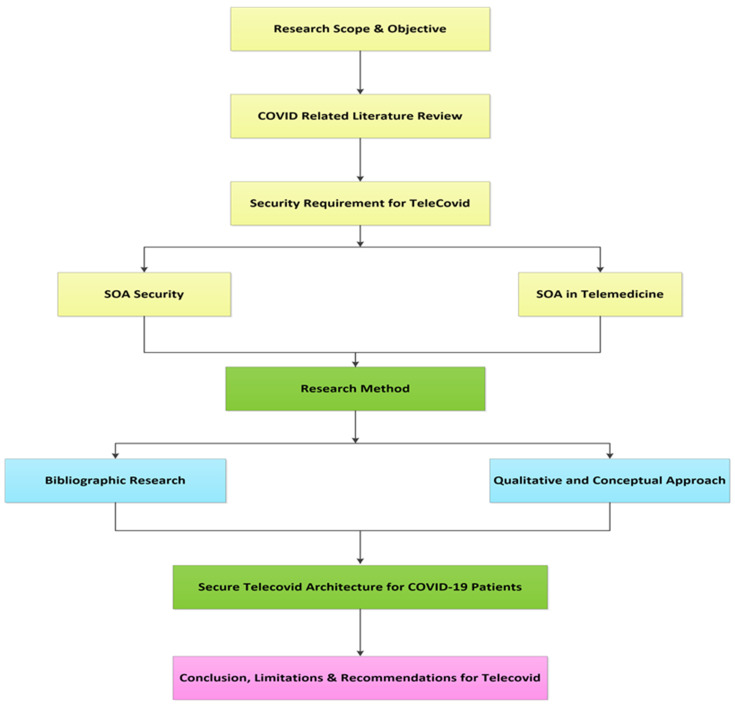
Flow chart of the proposed Tele-COVID architecture.

The flow chart is of the proposed architecture is divided into the following steps:

**i**  Research Scope and Objectives: A Tele-COVID secure system architecture that provides treatment for COVID-19 patients remotely while overcoming problems of data interchange problem that occurs when different telemedicine applications are presented.
**ii**  COVID-related Literature Review: An extensive review has been conducted where it is identified that there are a number of SOA security architectures but those are not used in telemedicine. Based on the literature, it is discovered that there a very few architectures in the field of telemedicine with SOA.
**iii**  Security Requirement for Tele-COVID: A broad investigation to discuss major secure compunction requirements while facing COVID-19 pandemic has been discussed that is further divided into two sections: (1) SOA Security, and (2) SOA telemedicine.
**iv**  Research Method: A broad research method has been conducted to investigate existing architecture with comparison to proposed secure architecture which is further divided into two sections: (1) Bibliographic research, and (2) Qualitative and Conceptual research.
**v**  Secure Tele-COVID Architecture for COVID-19 Patients: The presented architecture is designed to integrate service-oriented architecture while utilizing secure communication mode that prevents violation of patient privacy as well as ensuring secure, robust, and flexible characteristics is presented in this design.
**vi**  Conclusion, Limitations, and Recommendations for Tele-COVID: SOA-based architecture has been proposed to provide several web services as assistants for CIOVID-19 patients, on the other hand; limitations and recommendations are presented to be addressed in future work. Figure 4 shows the flow chart of the proposed Tele-COVID Architecture.

## 5. Proposed Security Architecture

**Figure 5:**
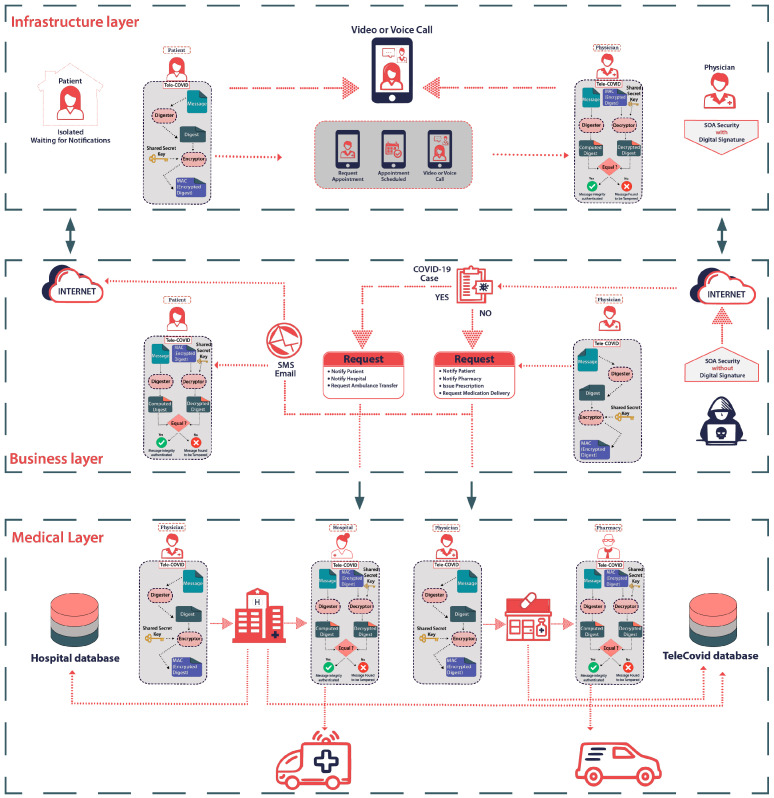
SOA Security architecture of Tele-COVID.

COVID-19 pandemic is spreading very fast and the current wave of the virus is called the third wave which is more dangerous than the first and second wave [27]. There is a difference between the first, second, and third waves of COVID-19 [28]. With the passage of time, COVID-19 waves are becoming more dangerous and the virus is changing its shape [29]. This shape change increases the infection and mortality rate and to overcome this situation, telemedicine applications can provide a robust solution. A number of telemedicine applications are installed and currently functioning in the hospitals but these systems are not particularly installed to treat COVID-19 patients. The architecture of Tele-COVID is designed differently with the integration of SOA using the secure mode of communication where the patient’s privacy is never compromised. In order to ensure a secure, robust, and flexible telemedicine system design, several existing systems are observed [30,31,32]. These systems propose multi-objective optimal medical (MooM) data processing technique using machine learning and deep learning techniques [33,34,35]. Consequently, it is discovered that there are a very limited number of SOA-based applications and none of them is used for COVID-19 patients. Figure 5 shows the SOA security architecture of the Tele-COVID application which is built with integration of Figure 2. The components of the Tele-COVID model are easily extendable to any running telemedicine application and the patient record can be transferred securely to any similar or dissimilar application. This resolves the problem of data inconsistency between two same or different applications. There are several challenges faced when there is a transfer of patients’ history from one application to another. Tele-COVID solves the problems of data integration, vendor lock-in, and interpretability. The target audience for the designed architecture is limited to COVID-19 patients. However, after some modification, it is possible to integrate Tele-COVID architecture for other applications.

The architecture is based on two interfaces of communication i.e., mobile interface, and web-based interface. Both interfaces are secured with encryption and decryption techniques using SSK. The communication can be established between physician and patient, physician and hospital, and physician and pharmacy with secure channels or mode of transmission. Global System for Mobile Communications/Fourth Generation (GSM/4G) telecommunication technology is used to establish the connection between mobile and web-based architectures. The communication is based on different messages and each message is consists of a token. The input is a value that can be patient ID, patient name, and the message, while the output is token initiation with an encrypted message. In case of wrong data provided, the token will be expired and a new token can be initiated for another message. Once the token is created successfully with correct patient data, the encrypted message will be generated, and the normal form of the text will be transformed into ciphertext. The ciphertext can be decrypted on the other end using the information available in the token. This process ensures secure communication between all ends in both interfaces of Tele-COVID. The following steps and Algorithm 1 shows the process of token initialization and message encryption.
**Algorithm 1** Initiate the token and encrypt the message     **Input** Values of the Patient ID, Patient Name, Message     **Output** Device token and encrypt message1:**procedure**Initiate the token2:    *Token ← Get an updated token and*3:                        *register it in app’s server*4:    **if** *token initiated* ≠ *NULL* **then**5:        $Patient_{ID}\leftarrow EnteredPatient_{ID}$6:        $Patient_{Name}\leftarrow EnteredPatient_{Name}$7:        Message← encrypt*(Entered Message)*8:        Send *(Entered Data)*9:    **else**10:        *Error Please enter the required data*11:    **end if**12:**end procedure**  13:**function**encrypt(Message)14:    **return** Cipher15:**end function**16:**function**Send(Data)17:    **return** *Send data to intended receiver*18:**end function**

1. Patient or care provider download Tele-COVID app.
2. If the device has not been registered before a token will be initiated for the device and registration will be completed in the app’s server.
3. If the device has initiated a token previously, then the initiated token will be retrieved from the server to establish secure communication.
4. The message will be encrypted and sent to the intended receiver.

Different layers of Open Systems Interconnection (OSI) models are considered that software systems use to communicate over a network. Tele-COVID design is based on three distinct layers, i.e., the business layer, infrastructure layer, and medical layer.

The data/information is transmitted between the physician and the patient through the infrastructure layer. The patient will acquire an appointment with the physician using Tele-COVID request appointment options. The secure 4G connection will be established after the formal approval of the appointment. There will be several doctors available via Tele-COVID to prescribe the treatment and provide medical assistance to COVID-19 patients. The doctor will take a symptom history from a patient and decide the appropriate treatment. In case of severity, the doctor can request a hospital to move the patient from home to the COVID ward. Alternatively, self-isolation can be advised if the condition of the patient is stable and having only mild symptoms. Simultaneously, the physician may write a medical prescription and the pharmacy will receive it through the Tele-COVID application.

The other layer is the business layer that establishes the communication between numerous modules such as 4G providers and external web services. Tele-COVID is implemented for web-based applications and smartphones with an Android mobile operating system. The programming of the web-based application is performed in Visual Studio.Net using an Internet Information Server (IIS). MySQL is used as a database. There is also a record and store option available for the physician once the communication is established between any end. This option will help a physician to explore patient history anytime to provide better medical assistance.

The medical layer demonstrates the transportation process when the patient needs medical support. The system is utilised with an Electronic Health Record (EHR) which can be established connecting between the hospital database and TeleCovid database. For instance, “InsertData” and “GetData” are two main services that are offered by the application. “InsertData” is developed to insert an entry into a database such as a physician prescription. However, “GetData” is used to retrieve the entry. The physician needs to log on to avail of these services and then the prescription details can be entered into Tele-COVID. Other heterogeneous telemedicine applications can also retrieve this information with certain limitations. Tele-COVID is fully capable to provide medical care to COVID patients from any part of the world and therefore, it improves the proficiency of medical care for the patients who cannot attend the doctor or move freely.

## 6. SOA Security Implementation in Tele-COVID

The word security can be classified as a sum of integrity, confidentiality, and privacy and it can be acquired by protecting from unauthorized access to the patient data. Several telemedicine applications are currently working to provide health services to patients in different parts of the world. However, the security for communication is compromised due to the diverse nature of these heterogeneous environments. Therefore, the data interchange between two similar or dissimilar telemedicine applications is a difficult task. Also, the message passing between patient and physician is not fully secure.

Tele-COVID is protected using digital signature, authentication, authorization, and encryption techniques. The security is implemented on two ends i.e., physician’s end, and patient’s end. The patients who are registered in Tele-COVID with their national ID and phone number can only request an appointment. The registration phase involves a code which will be sent to patients registered mobile number through Short Message Service (SMS). Once confirmed, the process of registration will begin.

The other part of the security implementation is the doctor’s end which is the most essential part and the chances of vulnerability are high. The framework is designed using Apache Axis [36]. Apache Axis is a web service system that is open source using Extensible Markup Language (XML). Moreover, Apache Axis security is a java based (WSS4J) that protects the execution of web services. WSS4J is used to authenticate SOAP messages with Web Services Security (WS-Security) data and it generates the SOAP message links with XML security, signature, and encryption. Furthermore, it creates tokens for timestamps, username, and SecurityAssertion Markup Language (SAML). Tele-COVID activities assure security through the initialization of username tokens. WSS4J regulators are introduced for the Web Service Deployment Descriptor (WSDD) to maintain with the WS-Security. Additionally, there are a couple of security regulators that approve and validate the request. SOAP messages can be decrypted with a public key and in ResponseFlow, each response will be recorded.

**Figure 6:**
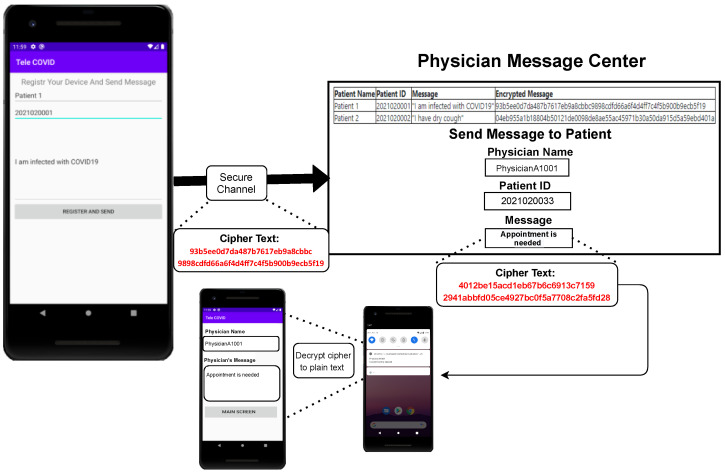
Tele-COVID messaging process from patient to physician.

Figure 6 shows the secure message passing from patient to physician. The patient will send a message using the mobile Tele-COVID application. This message will be converted automatically into ciphertext and the physician receives the message on the Physician Message Center (PMC). The physician will be able to decrypt the message using PMC and a reply can also be generated. The generated reply from the physician will also be transformed into ciphertext and can be delivered securely to the patient.

The main objective of the proposed Tele-COVID is to provide secure communication between patients and care providers. The Tele-COVID system will encrypt all messages between all entities utilizing the proposed system. Therefore, all messages will be encrypted using a unique token for each registered device to communicate securely. On the other hand, all messages sent from the care providers will be encrypted as well and they can be read and opened by the intended receiver only.

### Prevention from Reply Attacks

**Figure 7:**
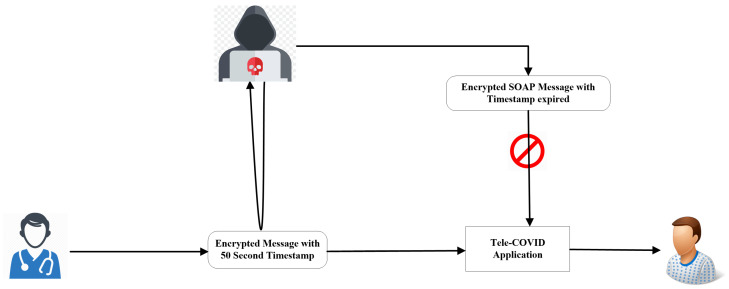
Tele-COVID security mechanism with a timestamp.

The most common type of attack is the “playback attack”, where attackers can create a connection to acquire patients’ data and transmission is maliciously or fraudulently repeated or delayed. In a playback attack, one can get access to encrypted SOAP messages and hack the patient’s personal medical history or information. Tele-COVID utilized timestamping to secure the message passing from one end to the other. Timestamping is based on certain strings showing the information and time when the service is requested. Timestamping records every service by a program. For every sent and received message using Tele-COVID, we set a length of 50 s for each timestamp. The message will be deleted when the timestamp is expired. Figure 7 provides a proposed security mechanism with a timestamp that can be used as a counteraction for a reply attack.

## 7. Discussions and Evaluation of Tele-COVID SOA Security Architecture

In this section, the advantages and limitations along with results are described. Tele-COVID Security architecture is SOA based, therefore, the major advantage of the design is the reuse of components between similar and dissimilar telemedicine applications. The reuse can be achieved without investing in expensive technologies. The communication protocol of the architecture systematizes the communication between Tele-COVID and external web services through WSDL. Comprehend XML contracts need to generate to disseminate information between two different telemedicine applications. The substance type from divergent telemedicine application will be identified through a web service metadata exchange facility which is implemented by default in Tele-COVID. With the help of these web services, the problem of data exchanges is resolved. Consequently, the services can be utilized for two different telemedicine applications developed by distinct vendors, and thus, vendor locking issues will no longer be crucial and communication can be established easily between two dissimilar applications. This concept removes the obligation of users for being locked to a single vendor. The services adopted by Tele-COVID are SOA and the implementation of the security is also SOA based.

There are several features of Tele-COVID that are convenient for COVID-19 patients:

1. The first option is a home test which is based on certain questionnaires. Once these questionnaires are filled by the suspected COVID-19 patients, the Tele-COVID telemedicine application will advise the patient to seek immediate medical assistance if required. Alternatively, home quarantine is advised. The home test feature is very useful for those patients who are sensitive and afraid to seek an appointment with a doctor.
2. The second option is to reserve an appointment in case Tele-COVID recommends taking the advice of the physician. The second option automatically will be enabled after the questionnaires are filled and the patient requires an appointment. The list of physicians becomes available as per the location of the patient. There is a possibility that the physician can be overbooked in a particular area. In that case, the nearest available physician will be assigned for medical advice. Each physician is capable and obliged of handling a certain number of patients per day.
3. The third option is to track medicine; this option is accessible to the patient if the physician has prescribed medication. The physician enters the medical prescription through Tele-COVID and the pharmacy is informed.
4. The final option is to order an ambulance in case the patient is serious and has a breathing problem. Ambulance service can also be tracked by the patient through Tele-COVID. This option can only be enabled by the physician and the ambulance will move the patient from home to the COVID-19 special treatment ward.

**Figure 8:**
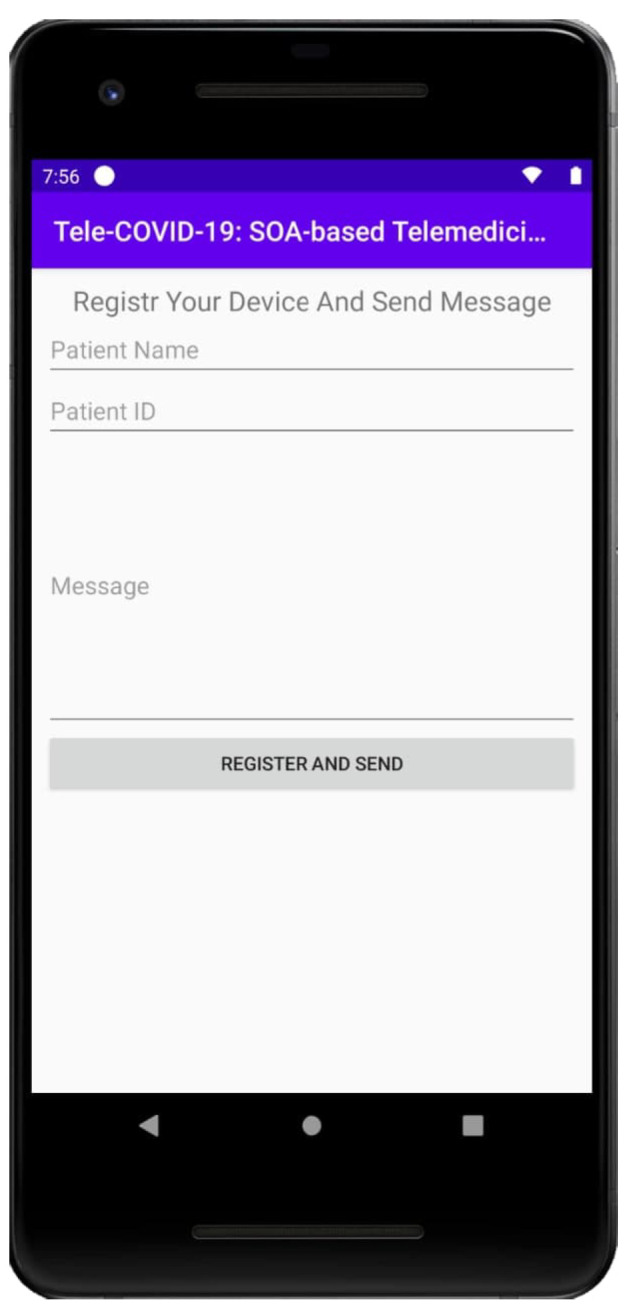
Tele-COVID patient registration interface.

**Figure 9:**
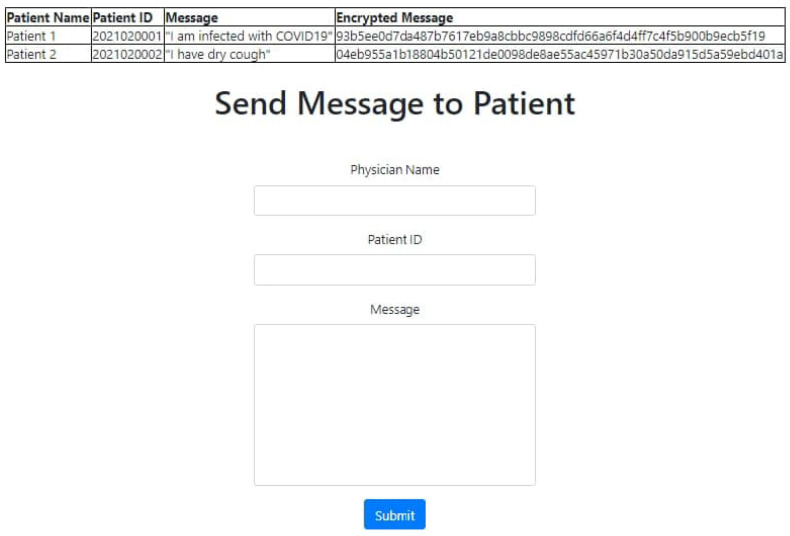
Tele-COVID physician response interface.

To conduct the experiments, Tele-COVID is deployed in the county hospital using all four parts, i.e., patent, doctor, hospital, and pharmacy. Figure 8 shows the main mobile application interface for patient registration while Figure 9 depicts a physician response system deployed in the hospital.

Table 1 shows experimental results for 25 patients where column Patient ID shows the identification/national ID number of the patient, columnPatient Name illustrates the name of the patient, column Physician ID demonstrates the hospital ID along with the physician name. Due to privacy concerns, all the above information is kept secret, and only dummy information is entered into the table for columns Patient ID, Patient name, Physician ID, and Physician Name. Similarly, the column Patient Request Date, Time, and Symptoms show the appointment requested and the reported symptoms from the patient to a physician. Moreover, column Connection Established Time demonstrated the conversation time and column Patient’s Encrypted Message shows an encrypted message transferred through Tele-COVID between the patient and the doctor.

Table 2 demonstrates the communication between the physician and the hospital after finalizing with each patient. All results are generated using Tee-COVID SOA secure application. The column Tele-COVID Response Time, and Diagnosis shows the action taken by the physician. For example, patient two in row two (Table 1) reported cough as a symptom, and after few hours physician prescribed medication for patient two in row two (Table 2). In addition, the column Physician’s Encrypted Message illustrates the encrypted message sent to the patient, hospital, or pharmacy. Column Hospital Response Time and Message indicates the response time to the physician. All services in Tele-COVID provide rapid response. For example, the ambulance was requested for ‘patient 0104’ on “Tues 5 Jan. 2021 11:13 a.m.” and was dispatched on “Tues 5 Jan. 2021 12:01 p.m.” within 50 min. Patient 0104 reported “My symptoms for COVID are that difficulty breathing and shortness of breath”, and therefore, immediate medical assistance was provided. The column Hospital’s Encrypted Message demonstrates a secured message sent to the physician. For example, column Ambulance Dispatched shows the time and message sent to a physician that an ambulance is departed to transport the patient P 0104. Finally, column Pharmacy Response Time and Message and column Pharmacy’s Encrypted Message illustrate the response time and secured message sent to a physician for each patient.

SOA plays a vital role in delivering the services used in telemedicine architectures. Since the developed architecture is SOA-based, the primary advantage is the reuse of the components between several organizations without implementing any new and expensive technologies. With the help of WSDL, communication can be standardized between Tele-COVID and external web services. To establish successful communication among two dissimilar telemedicine applications, both ends require an understanding of XML to collaborate information at different stages. To identify the data and its subcomponents from different telemedicine applications, there is a need for web service metadata exchange. By employing several web services, it is easier and convenable to identify and solve the problem of data exchange. Alternatively, two distinct telemedicine applications from different vendors can utilize these services and the applications can transmit the data without any interruption. With this concept, the users are not dependent on a single vendor. Every single service offered by Tele-COVID is SOA-based and the results shown in Table 1 and Table 2 are achieved by utilizing SOA features.

The limitation of Tele-COVID security architecture is the transmission of data through semantic interoperability. There will be successful data transfer between two dissimilar Tele-COVID applications in the case of semantic interoperability. Semantic interoperability is the capability of computer systems to transmit the data with explicit meaning. Semantic interoperability exploits both the organizing of the information exchange and the codification of the information including vocabulary with the goal that the receiving data frameworks can interpret the information.

## 8. Conclusions and Future Work

Telemedicine applications are being adopted widely to treat COVID-19 patients as they provide treatment to patients without having them explicitly pay a visit to the hospital. Self-isolation is mandatory for most COVID-19 patients and therefore with the help of telemedicine applications, appropriate medical assistance can be provided. SOA offers different services, and it can facilitate with privileged benefits designed for vendors, products, and technologies. An SOA-based architectural design named Tele-COVID is proposed in this paper, which consists of several web services to provide medical assistant for COVID-19 patients. There is a total of four SOA-based services discussed in this paper and each service is uniquely featured. The purpose of each service is to facilitate COVID-19 patients without having them leave their residence. Tele-COVID architectural design is fully secured and can protect patients’ and doctors’ history from any type of cyber-attack. Tele-COVID features are distinctively programmed so that the data of patients and medical staff can be transferred from one telemedicine application to other.

As our future work, we plan to explore two research directions. First, we aim to investigate semantic interoperability, which can still transmit the data in different data structures. Second, extending SOA services with cloud computing and the Internet of things (IoT) to adopt more convenient web and Android services.

## Figures and Tables

**Table 1:** Tele-COVID patient-physician communication data in the county hospital.

No	Patient ID	Patient Name	Physician ID	Physician Name	Patient Request Date, Time and Symptoms	Connection Established Time	Patient’s Encrypted Message
1	Patient0101	Patient 1	PhysicianA1001	Physician A	Mon 4 Jan. 2021 7:24 a.m. I am infected with COVID-19	Mon 4 Jan. 2021 11:51 a.m.	Encrypted Message Here
2	Patient0102	Patient 2	PhysicianA1001	Physician A	Mon 4 Jan. 2021 9:06 a.m. I have notice that my cough is too dry	Mon 4 Jan. 2021 13:33 p.m.	Encrypted Message Here
3	Patient0103	Patient 3	PhysicianA1001	Physician A	Mon 4 Jan. 2021 10:17 a.m. Recently, I just loss of taste and barely smell	Mon 4 Jan. 2021 13:51 p.m.	Encrypted Message Here
4	Patient0104	Patient 4	PhysicianA1001	Physician A	Mon 4 Jan. 2021 11:35 a.m. My symptoms for COVID are that difficulty breathing and shortness of breath	Mon 4 Jan. 2021 14:08 p.m.	Encrypted Message Here
5	Patient0105	Patient 5	PhysicianA1001	Physician A	Mon 4 Jan. 2021 14:45 a.m. I have a loss of speech and movement	Mon 4 Jan. 2021 15:12 p.m.	Encrypted Message Here
6	Patient0106	Patient 6	PhysicianB1002	Physician B	Tues 5 Jan. 2021 8:16 a.m. I have a chest pain and pressure	Tues 5 Jan. 2021 09:17 a.m.	Encrypted Message Here
7	Patient0107	Patient 7	PhysicianB1002	Physician B	Tues 5 Jan. 2021 9:04 a.m. I have a strange headache	Tues 5 Jan. 2021 09:21 a.m.	Encrypted Message Here
8	Patient0108	Patient 8	PhysicianB1002	Physician B	Tues 5 Jan. 2021 9:16 a.m. I have a conjunctivitis	Tues 5 Jan. 2021 10:26 a.m.	Encrypted Message Here
9	Patient0109	Patient 9	PhysicianB1002	Physician B	Tues 5 Jan. 2021 11:36 a.m. I have a saver corona Symptoms	Tues 5 Jan. 2021 11:53 a.m.	Encrypted Message Here
10	Patient0110	Patient 10	PhysicianB1002	Physician B	Tues 5 Jan. 2021 11:52 a.m. I have a fever, chest pain and loss of taste	Tues 5 Jan. 2021 14:16 p.m.	Encrypted Message Here
11	Patient0111	Patient 11	PhysicianC1003	Physician C	Wend 6 Jan. 2021 11:24 a.m. I feel aches and pains this morning	Wend 6 Jan. 2021 15:05 p.m.	Encrypted Message Here
12	Patient0112	Patient 12	PhysicianC1003	Physician C	Wend 6 Jan. 2021 11:32 a.m. Nasal congestion and runny nose. I need to see a doctor	Wend 6 Jan. 2021 15:25 p.m.	Encrypted Message Here
13	Patient0113	Patient 13	PhysicianC1003	Physician C	Wend 6 Jan. 2021 12:13 p.m. I am not sure if I am infected with COVID. Any consultation appointment?	Wend 6 Jan. 2021 15:45 p.m.	Encrypted Message Here
14	Patient0114	Patient 14	PhysicianC1003	Physician C	Wend 6 Jan. 2021 13:41 p.m. I am unable to speech and move	Wend 6 Jan. 2021 16:07 p.m.	Encrypted Message Here
15	Patient0115	Patient 15	PhysicianC1003	Physician C	Wend 6 Jan. 2021 14:14 p.m. I am experiencing lately some tiredness and fatigueness	Wend 6 Jan. 2021 16:19 p.m.	Encrypted Message Here
16	Patient0116	Patient 16	PhysicianD1004	Physician D	Thurs 7 Jan. 2021 10:05 a.m. Serious signs of covid19 which affect my living during the day	Thurs 7 Jan. 2021 11:03 a.m.	Encrypted Message Here
17	Patient0117	Patient 17	PhysicianD1004	Physician D	Thurs 7 Jan. 2021 10:43 a.m. I was in a room with coronavirus infected person	Thurs 7 Jan. 2021 11:17 a.m.	Encrypted Message Here
18	Patient0118	Patient 18	PhysicianD1004	Physician D	Thurs 7 Jan. 2021 10:54 a.m. I started to feel some fever	Thurs 7 Jan. 2021 11:33 a.m.	Encrypted Message Here
19	Patient0119	Patient 19	PhysicianD1004	Physician D	Thurs 7 Jan. 2021 11:36 a.m. My partner has body pains for a week	Thurs 7 Jan. 2021 11:59 a.m.	Encrypted Message Here
20	Patient0120	Patient 20	PhysicianD1004	Physician D	Thurs 7 Jan. 2021 15:58 a.m. I am a 73-year person, and I was diagnosed with COVID 2 month ago	Thurs 7 Jan. 2021 16:14 p.m.	Encrypted Message Here
21	Patient0121	Patient 21	PhysicianE1005	Physician E	Sun 7 Feb. 2021 9:16 a.m. I have a shortness of breath when I talk or walk	Sun 7 Feb. 2021 11:25 a.m.	Encrypted Message Here
22	Patient0122	Patient 22	PhysicianE1005	Physician E	Tues 9 Feb. 2021 12:42 p.m. I feel strong headache for a quite long period of time	Tues 9 Feb. 2021 1:18 p.m.	Encrypted Message Here
23	Patient0123	Patient 23	PhysicianE1005	Physician E	Wend 10 Feb. 2021 11:27 a.m. Unable to breath and I have side effects of some drugs for covid	Wend 10 Feb. 2021 11:57 a.m.	Encrypted Message Here
24	Patient0124	Patient 24	PhysicianE1005	Physician E	Thurs 11 Feb. 2021 9:48 a.m. I think I am infected with COVID because I have a diarrhoea	Thurs 11 Feb. 2021 10:21 a.m.	Encrypted Message Here
25	Patient0125	Patient 25	PhysicianE1005	Physician E	Thurs 11 Feb. 2021 11:59 a.m. I have a sore throat	Thurs 11 Feb. 2021 15:37 p.m.	Encrypted Message Here

**Table 2:** Tele-COVID physician-hospital communication data in the county hospital.

Tele-COVID Response Time, and Diagnosis	Physician’s Encrypted Message	Hospital Response Time and Message	Hospital’s Encrypted Message	Ambulance Dispatched	Pharmacy Response Time and Message	Pharmacy’s Encrypted Message
Tues 5 Jan. 2021 9:07 a.m. Appointement is needed	Encrypted Message Here	Tues 5 Jan. 2021 09:26 a.m.	Not Applicable	Not Applicable	Not Applicable	Not Applicable
Tues 5 Jan. 2021 10:04 a.m. Medicine is prescribed	Not Applicable	Tues 5 Jan. 2021 10:16 a.m.	Not Applicable	Not Applicable	Thurs 7 Jan. 2021 14:41 p.m.	Encrypted Message Here
Tues 5 Jan. 2021 10:28 a.m. Appointment is needed	Encrypted Message Here	Tues 5 Jan. 2021 10:55 a.m.	Not Applicable	Not Applicable	Not Applicable	Not Applicable
Tues 5 Jan. 2021 11:13 a.m. An a.m.bulance is sent	Not Applicable	Tues 5 Jan. 2021 11:24 a.m.	Encrypted Message Here	Tues 5 Jan. 2021 12:01 p.m.	Not Applicable	Not Applicable
Tues 5 Jan. 2021 13:02 p.m. Appointment is needed	Encrypted Message Here	Tues 5 Jan. 2021 13:43 p.m.	Not Applicable	Not Applicable	Not Applicable	Not Applicable
Wend 6 Jan. 2021 09:57 a.m. An a.m.bulance is sent	Not Applicable	Wend 6 Jan. 2021 10:03 a.m.	Encrypted Message Here	Wend 6 Jan. 2021 10:43 a.m.	Not Applicable	Not Applicable
Wend 6 Jan. 2021 10:33 a.m. Medicine is prescribed	Not Applicable	Wend 6 Jan. 2021 10:48 a.m.	Not Applicable	Not Applicable	Thurs 7 Jan. 2021 13:14 p.m.	Encrypted Message Here
Wend 6 Jan. 2021 11:07 a.m. Appointment is needed	Encrypted Message Here	Wend 6 Jan. 2021 11:34 a.m.	Not Applicable	Not Applicable	Not Applicable	Not Applicable
Wend 6 Jan. 2021 13:04 p.m. An a.m.bulance is sent	Not Applicable	Wend 6 Jan. 2021 13:19 p.m.	Encrypted Message Here	Wend 6 Jan. 2021 13:56 p.m.	Not Applicable	Not Applicable
Wend 6 Jan. 2021 13:43 p.m. An a.m.bulance is sent	Not Applicable	Wend 6 Jan. 2021 13:59 p.m.	Encrypted Message Here	Wend 6 Jan. 2021 15:16 p.m.	Not Applicable	Not Applicable
Thurs 7 Jan. 20219:33 a.m. Medicine is prescribed	Not Applicable	Thurs 7 Jan. 2021 10:07 a.m.	Not Applicable	Not Applicable	Fri 8 Jan. 2021 10:43 p.m.	Encrypted Message Here
Thurs 7 Jan. 2021 11:20 a.m. Appointment is needed	Encrypted Message Here	Thurs 7 Jan. 2021 11:37 a.m.	Not Applicable	Not Applicable	Not Applicable	Not Applicable
Thurs 7 Jan. 2021 14:10 p.m. Appointment is needed	Encrypted Message Here	Thurs 7 Jan. 2021 14:16 p.m.	Not Applicable	Not Applicable	Not Applicable	Not Applicable
Thurs 7 Jan. 2021 15:13 a.m. An a.m.bulance is sent	Not Applicable	Thurs 7 Jan. 2021 15:36 p.m.	Encrypted Message Here	Thurs 7 Jan. 2021 15:47 p.m.	Not Applicable	Not Applicable
Thurs 7 Jan. 2021 15:50 p.m. Medicine is prescribed	Not Applicable	Thurs 7 Jan. 2021 16:24 p.m.	Not Applicable	Not Applicable	Fri 8 Jan. 2021 14:42 p.m.	Encrypted Message Here
Fri 8 Jan. 2021 9:37 a.m. An a.m.bulance is sent	Not Applicable	Fri 8 Jan. 2021 10:11 a.m.	Encrypted Message Here	Fri 8 Jan. 2021 10:52 a.m.	Not Applicable	Not Applicable
Fri 8 Jan. 2021 10:17 a.m. Appointment is needed	Encrypted Message Here	Fri 8 Jan. 2021 10:39 a.m.	Not Applicable	Not Applicable	Not Applicable	Not Applicable
Fri 8 Jan. 2021 10:53 a.m. Medicine is prescribed	Not Applicable	Fri 8 Jan. 2021 11:18 a.m.	Not Applicable	Not Applicable	Sat 9 Jan. 2021 14:49 p.m.	Encrypted Message Here
Fri 8 Jan. 2021 11:25 a.m. Medicine is prescribed	Not Applicable	Fri 8 Jan. 2021 11:47 a.m.	Not Applicable	Not Applicable	Sat 9 Jan. 2021 14:49 p.m.	Encrypted Message Here
Fri 8 Jan. 2021 14:10 p.m. Appointment is needed	Encrypted Message Here	Fri 8 Jan. 2021 14:34 p.m.	Not Applicable	Not Applicable	Not Applicable	Not Applicable
Mon 8 Feb. 2021 19:07 a.m. An a.m.bulance is sent	Not Applicable	Mon 8 Feb. 2021 14:39 a.m.	Encrypted Message Here	Mon 8 Feb. 2021 20:32 a.m.	Not Applicable	Not Applicable
Tues 9 Feb. 2021 15:22 p.m. Appointment is needed	Encrypted Message Here	Wend 10 Feb. 2021 10:09 p.m.	Not Applicable	Not Applicable	Not Applicable	Not Applicable
Wend 10 Feb. 2021 13:17 p.m. An ambulance is sent	Not Applicable	Wend 10 Feb. 2021 13:48 p.m.	Encrypted Message Here	Wend 10 Feb. 2021 21:19 p.m.	Not Applicable	Not Applicable
Thurs 11 Feb. 2021 12:40 p.m. Appointment is needed	Encrypted Message Here	Thurs 11 Feb. 2021 14:11 p.m.	Not Applicable	Not Applicable	Not Applicable	Not Applicable
Fri 12 Feb. 2021 09:19 a.m. Medicine is prescribed	Not Applicable	Fri 12 Feb. 2021 10:48 a.m.	Not Applicable	Not Applicable	Fri 12 Feb. 2021 11:15 a.m.	Encrypted Message Here
